# Clinical and endoscopic features of aorto-duodenal fistula resulting in its definitive diagnosis: an observational study

**DOI:** 10.1186/s12876-021-01616-9

**Published:** 2021-02-01

**Authors:** Chikamasa Ichita, Akiko Sasaki, Chihiro Sumida, Karen Kimura, Takashi Nishino, Junichi Tasaki, Sakue Masuda, Kazuya Koizumi, Jun Kawachi, Makoto Kako

**Affiliations:** 1 Gastroenterology Medicine Center, Shonankamakura General Hospital, 1370-1 Okamoto, Kamakura, Kanagawa 247-8533 Japan; 2 Department of Surgery, Shonan Kamakura General Hospital, Kamakura, Kanagawa Japan

**Keywords:** Aorto-duodenal fistula, Aorto-enteric fistula, Upper gastrointestinal bleeding

## Abstract

**Background:**

Upper gastrointestinal (GI) bleeding is the most important presentation of an aorto-duodenal fistula (ADF). Early diagnosis is difficult, and the disease is associated with high mortality. The present study aimed to examine the clinical and the endoscopic characteristics of ADF in eight patients who presented to our hospital. We also sought to clarify the diagnostic approach towards the disease.

**Methods:**

The present study examined the clinical and the endoscopic/computed tomography (CT) characteristics of ADF in eight patients who were definitively diagnosed with this condition in a 12-year period at our hospital.

**Results:**

The patients comprised of five men and three women, with a mean age of 69.8 years. Upper gastrointestinal bleeding was the chief complaint for all the patients. Out of these, two patients presented with shock. The patients’ mean haemoglobin at presentation was 7.09 g/dL, and the mean number of blood transfusions was 7.5. All patients had undergone intervention to manage an aortic pathology in the past. As the first investigation, an upper GI endoscopy in 5 and a CT scan in 3 patients were performed. In cases where CT scan was performed first, no definitive diagnosis was obtained, and the diagnosis was confirmed by performing an upper GI endoscopy. In cases where endoscopy was performed first, definitive diagnosis was made in only one case, and the other cases were confirmed by the CT scan. In some cases, tip attachments, converting to long endoscopes, and marking clips were found useful.

**Conclusions:**

In patients who have undergone intervention to manage an aortic pathology and have episodes of upper gastrointestinal bleeding, ADF cannot be definitively diagnosed with only one investigation. In addition, when performing upper GI endoscopy in cases where an ADF is suspected, tip attachment, converting to a long endoscope, and using marking clips can be helpful.

## Background

Aorto-duodenal fistula (ADF) is found in 0.05–0.07% of autopsies and is, therefore, considered rare [1]. It is difficult to diagnose early and is associated with high mortality [2]. Few case reports of ADF have been published, although some studies have summarised multiple cases [1–17]. Our institution is an emergency hospital that was able to accommodate at least 14,000 emergency transports and at least 43,000 emergency consultations in 2019. As such, a relatively high number of cases of ADF have been treated in our institution. The present study aimed to examine the clinical and endoscopic characteristics of ADF in eight patients at our hospital. It also sought to clarify how the disease can be diagnosed appropriately.

## Methods

Eight patients were diagnosed with ADF at our institution between April 2009 and April 2020. The present study examined the baseline, clinical and laboratory data, past medical history, endoscopic and computed tomograohy (CT) scan findings, number of blood transfusions received, definitive treatment, and outcomes. The type of scope used during endoscopy, the use of tip attachments, and marking clips were also recorded. Shock was defined as systolic blood pressure below 60 mmHg. An increase in body temperature (> 38 °C), white blood cell (WBC) count, and C-reactive protein (CRP) level indicated the possibility of infection. We first performed endoscopy using GIF-Q260J (Olympus, Tokyo, Japan). However, when no obvious bleeding was found and bleeding from the deep duodenum (such as patients with a history of aortic pathology) was suspected, a detailed examination of the duodenum was performed using a tip attachment. When no abnormalities were found with using a tip attachment, we switched to a paediatric colonoscope PCF-PQ260L (Olympus, Tokyo, Japan) and searched for bleeding in the deep duodenum.

In this study, the diagnosis of ADF was made following the recommendations of a previous report [2–4, 6–11]. Endoscopic findings confirmed ADF when endoscopy showed an exposed aortic stent in the duodenum. In addition, ADF was suspected when pulsatile lesions (erosions, protuberances, or blood clots) were found in the duodenum without any other bleeding source, or when a massive amount of fresh blood from an unknown origin was observed in the duodenum [3–6]. For the CT findings, ADF was confirmed when CT showed extravascular leakage from the aorta to the duodenum. In addition, ADF was suspected when the aorta and the duodenum were adjacent to each other, ectopic air in the aorta or a saccular aneurysm of the aorta adjacent to the duodenum was present [2, 4, 7–11]. If ADF was suspected after one investigation, the other was performed. The findings of both the investigations were corroborated with each other to confirm the diagnosis. Surgery was promptly performed when the diagnosis was confirmed. In the case of upper gastrointestinal bleeding, our hospital’s protocol prioritises performing an upper GI endoscopy over a CT scan. CT examination was performed first only when there was a history of a recent endoscopy and when CT was more easily accessible.

## Results

The characteristics of patients with ADF are shown in Tables 1, 2, and 3. The patients comprised five men and three women, with a mean age of 69.8 years. Their chief complaints were haematemesis (three patients), dark stools (five patients), shock (two patients), syncope (one patient), and weakness (one patient). Of the six patients who presented with no signs of shock, two developed shock during endoscopy. One of them died as a result of uncontrolled bleeding during endoscopy. The mean haemoglobin level at presentation was 7.09 g/dL, and the mean number of blood transfusions was 7.5. All patients had undergone intervention to manage aortic pathology (7 months to 13 years prior): four underwent aortic stent implantation to manage an abdominal aortic aneurysm, three had vascular prosthesis replacement, and one underwent para-aortic radiation because of lymph node metastasis secondary to cervical cancer.

**Table 1:** Demographic and clinical data of eight patients

April 2009 to April 2020: n = 8 patients	
Age	69.8 years (41–76 years)
Sex	5 males, 3 females
Chief complaint	Hematemesis: 3 Black stool: 5 Shock: 2 Syncope: 1 Weakness: 1
Shock at presentation	2 (25%)
Mean hemoglobin at presentation	7.09 g/dL (5.6–9.4 g/dL)
Mean no. of blood transfusions	7.5 (4–14)
History of treatment for any aortic pathology	8 (100%)
Abdominal aortic aneurysm	
After stent placement	4 (50%)
After artificial blood vessel replacement	3 (37.5%)
After periaortic irradiation	1 (12.5%)
Endoscopic findings	
Stent exposed	1 (12.5%)
Pulsatile lesions (erosion, granulation, blood clots)	4 (50%)
Massive fresh bleeding of unknown origin in the duodenum identified	3 (37.5%)
Patients undergoing CT scan before endoscopy	3 patients
CT findings	
Aorta and duodenum adjacent to each other	3/3 (100%)
Air in the aorta adjacent to the duodenum	2/3 (66.7%)
Patients undergoing CT scan after endoscopy	4 patients
CT findings	
Aorta and duodenum adjacent to each other	3/3 (100%)
Extravascular leakage from the aorta to the duodenum	2/3 (66.7%)
Ectopic air in the aorta adjacent to the duodenum	4 patients
Saccular aneurysm adjacent to the duodenum	3/3 (100%)
Fistula site	Horizontal part: 7 Descending part: 1
Type of endoscope used in diagnosis	GIF-Q260J: 6 PCF-PQ260L: 2
Possible surgical treatment	7 (87.5%)
Outcome	Death during endoscopy: 1 (12.5%) Surgical treatment and survival: 7 (87.5%)

*CT* computed tomography

**Table 2:** Clinical characteristics of each ADF patient

Case	Age	Chief complaint	Shock at presentation	Hemoglobin at presentation (g/dL)	Body temperature (℃)	WBC (/μL)	CRP (mg/dL)	History of treatment for any aortic pathology	Comorbidities	Antithrombotic drug NSAIDs	No. of blood transfusions	Type of endoscope used for diagnosis	Use of tip attachment	Shock during endoscopy
1	60 s	Black stool/syncope	None	6.8	36.2	4900	1.33	AAA burst EVAR (9 years prior)	CKD	None	4	PCF-PQ260L	Yes	None
2	70 s	Black stool	None	7.2	36	9100	0.4	AAA EVAR (1 year prior)	None	None	4	GIF-Q260J	None	None
3	70 s	Vomiting	Yes	7.6	36.1	13,300	0.19	AAA vascular prosthesis replacement (3 years prior)	Angina CKD on HD	Aspirin ticlopidine	6	GIF-Q260J	None	None
4	70 s	Vomiting	None	7.5	35.5	7100	0.14	Dissecting aortic aneurysm TEVAR + vascular prosthesis replacement (2 years prior)	Angina CKD	Aspirin	10	GIF-Q260J	Yes	Yes → expired
5	40 s	Vomiting	Yes	5.6	35.3	17,400	9.11	Para-aortic lymph node irradiation (2 years prior)	Cervical cancer	Loxoprofen	8	GIF-Q260J	Yes	None
6	70 s	Black stool	None	9.4	36.6	11,200	1.06	AAA vascular prosthesis replacement (13 years prior)	CKD	None	8	GIF-Q260J	None	None
7	70 s	Black stool	None	6.4	37.3	15,500	14.9	AAA EVAR (5 years prior)	CKD Prostate cancer	None	6	GIF-Q260J	Yes	None
8	70 s	Black stool/weaknes	None	6.2	37.1	8000	7.94	AAA vascular prosthesis replacement (7 months prior)	Myocardial infarction	None	14	PCF-PQ260L	Yes	Yes

*WBC* white blood cell, *CRP* C-reactive protein, *AAA* abdominal aortic aneurysm, *EVAR* endovascular aneurysm repair, *TEVAR* thoracic endovascular aortic repair, *CKD* chronic kidney disease, *HD* haemodialysis, *NSAID* non-steroidal anti-inflammatory drug

**Table 3:** Endoscopic/CT findings and treatment in each ADF patient

Case	Endoscopic findings	Marking clip	CT Findings	Fistula site (part of duodenum)	Treatment	Outcome
1	Pulsatile granule-like protuberance of the duodenum	+	The aorta and duodenum were adjacent Ectopic air in the aorta	3rd	Vascular prosthesis replacement Omental patch closure Duodenal repair	Survived
2	Stent exposed into the duodenum	−	–	2nd	Vascular prosthesis replacement Jejunal patch	Survived
3	Massive fresh blood of unknown origin in the duodenum	−	The aorta and duodenum were adjacent Ectopic air in the aorta	3rd	Emergency EVAR	Survived
4	Massive fresh blood of unknown origin in the duodenum	−	(postmortem imaging) The aorta and duodenum were adjacent A saccular aneurysm of the aorta adjacent to the duodenum	3rd	None	Expired
5	Pulsatile lesion with blood clots in the duodenum	+	The aorta and duodenum were adjacent Extravascular leakage from the aorta to the duodenum	3rd	Emergency EVAR	Survived
6	Pulsatile lesion eroding into the duodenum	+	The aorta and duodenum were adjacent A saccular aneurysm of the aorta adjacent to the duodenum	3rd	Emergency EVAR	Survived
7	Pulsatile granule-like protuberance of the duodenum	+	The aorta and duodenum were adjacent Ectopic air in the aorta	3rd	Bilateral axillo-femoral bypass surgery Laparoscopic removal of vascular prosthesis	Survived
8	Massive fresh blood of unknown origin in the duodenum	−	The aorta and duodenum were adjacent	3rd	Emergency EVAR	Survived

*CT* computed tomography, *2nd* second portion of duodenum, *3rd* third portion of duodenum, *EVAR* endovascular aneurysm repair

**Fig. 1:**
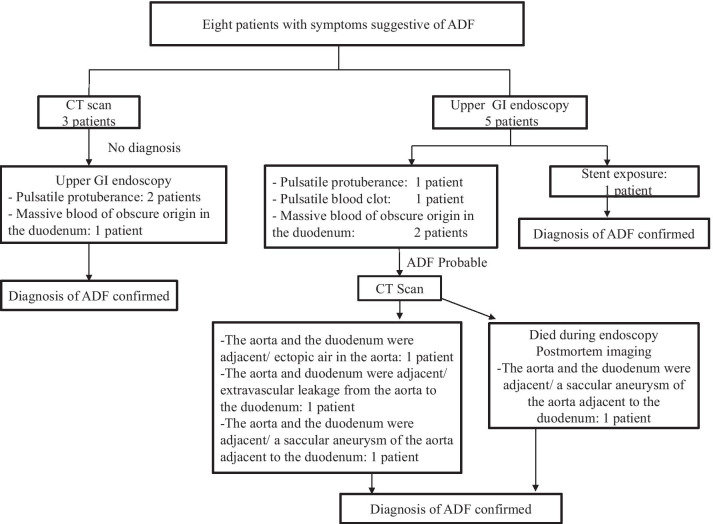
Flow chart showing diagnosis of aorto-duodenal fistula. *ADF* Aortoduodenal fistula, *CT* computed tomography

**Fig. 2:**
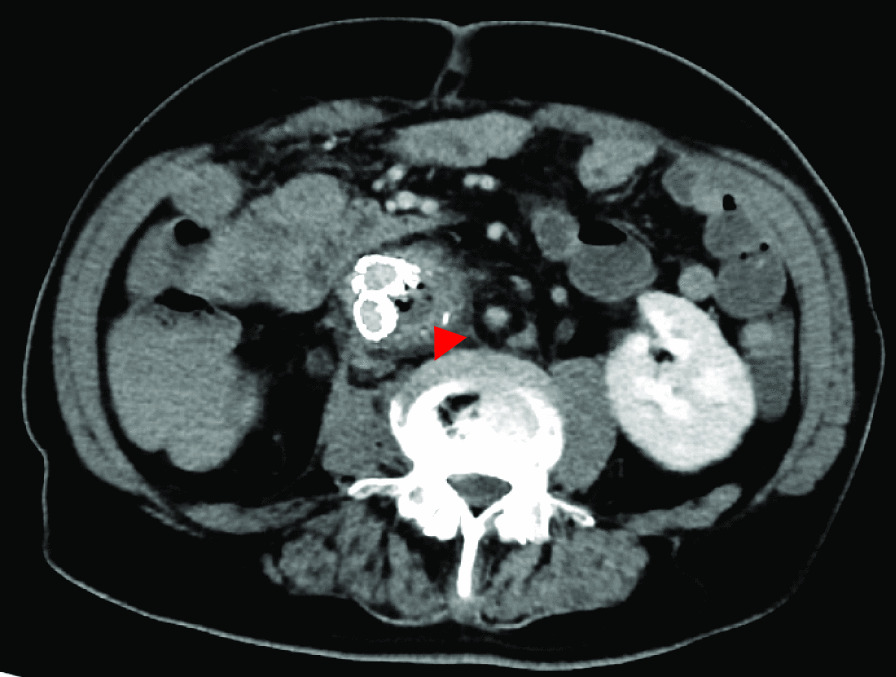
Contrast computed tomography image after upper endoscopy (case 2). Arrowhead: Image of air in the aorta

**Fig. 3:**
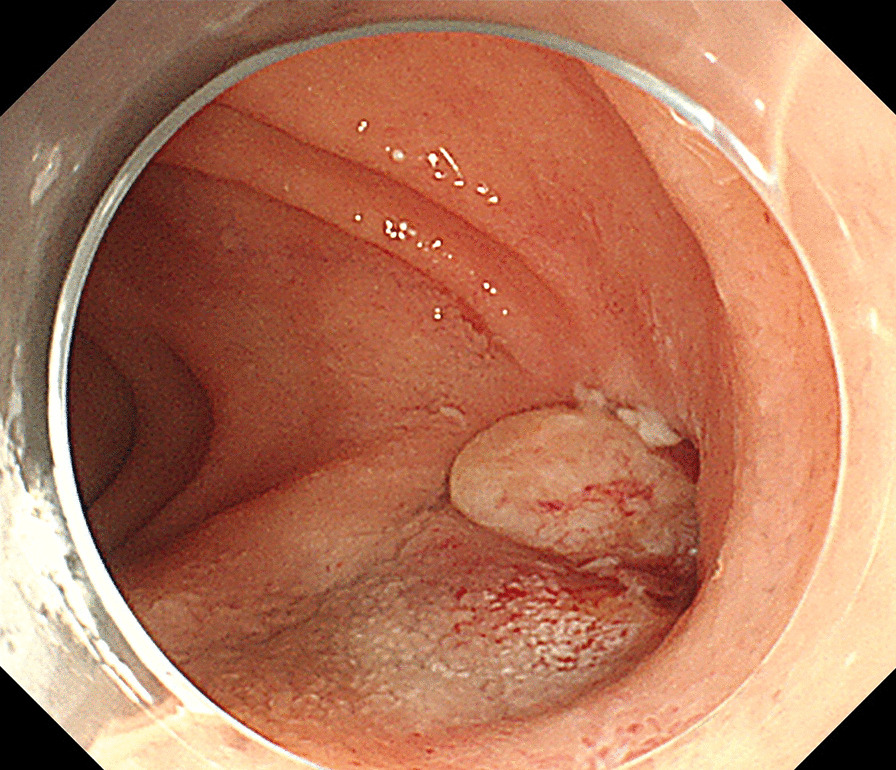
Upper endoscopy (case 1). A pulsatile, granulation-like protuberance in the 3rd portion of the duodenum

**Fig. 4:**
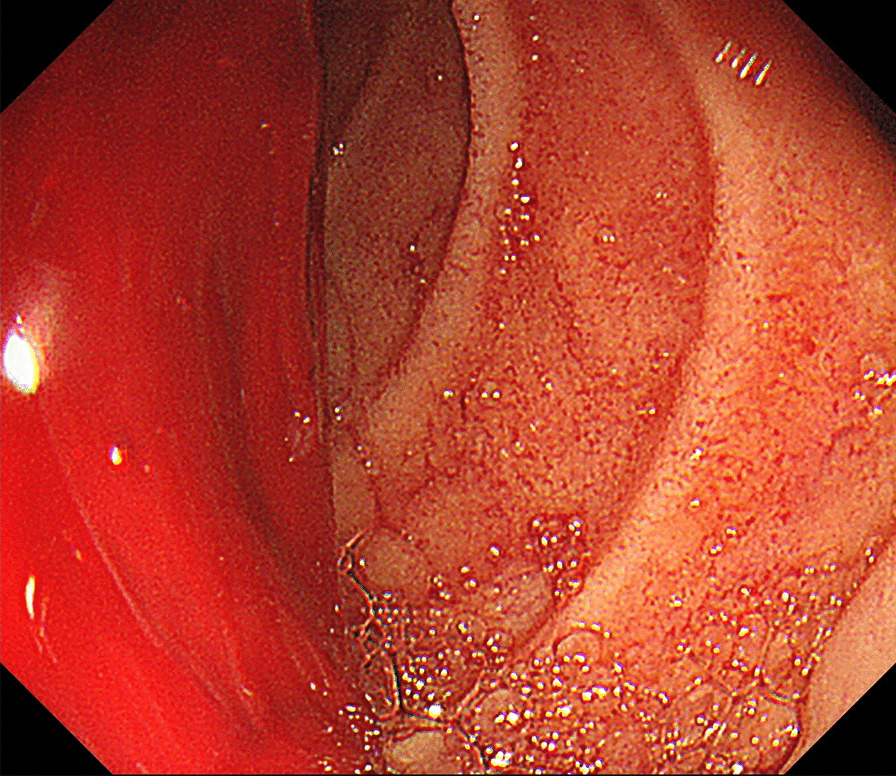
Upper endoscopy (case 8). Massive fresh bleeding of obscure origin is observed up to the 3rd portion of the duodenum

**Fig. 5:**
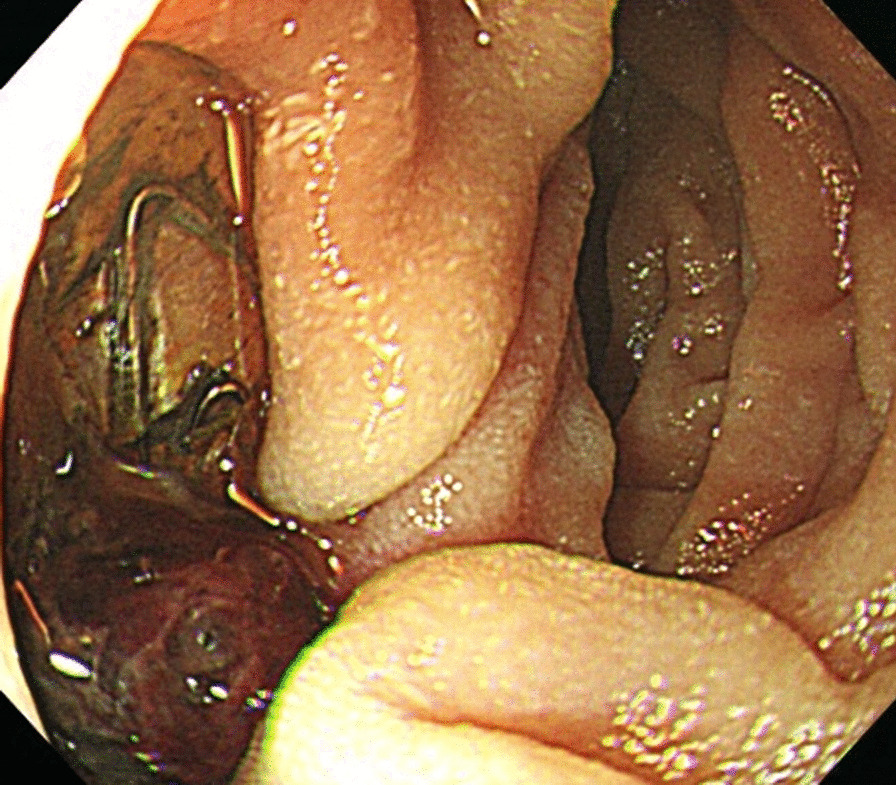
Upper endoscopy (case 2). Exposure of an aortic stent in the 2nd portion of the duodenum

**Fig. 6:**
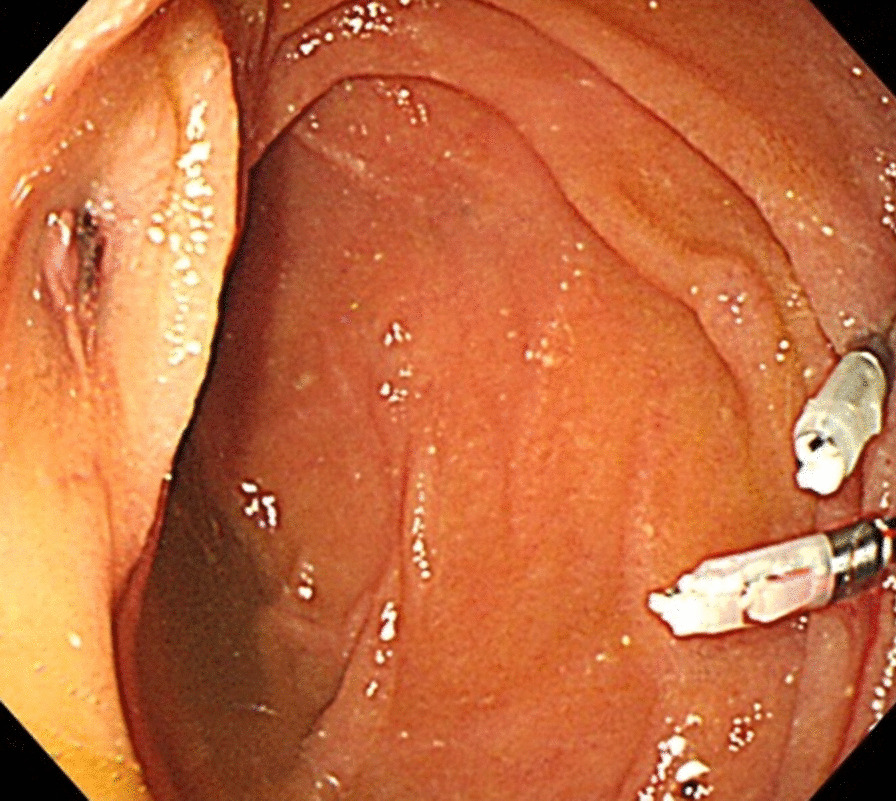
Upper endoscopy (case 6). Pulsatile erosion in the 3rd portion of the duodenum. A marking clip is placed on the opposite side

**Fig. 7:**
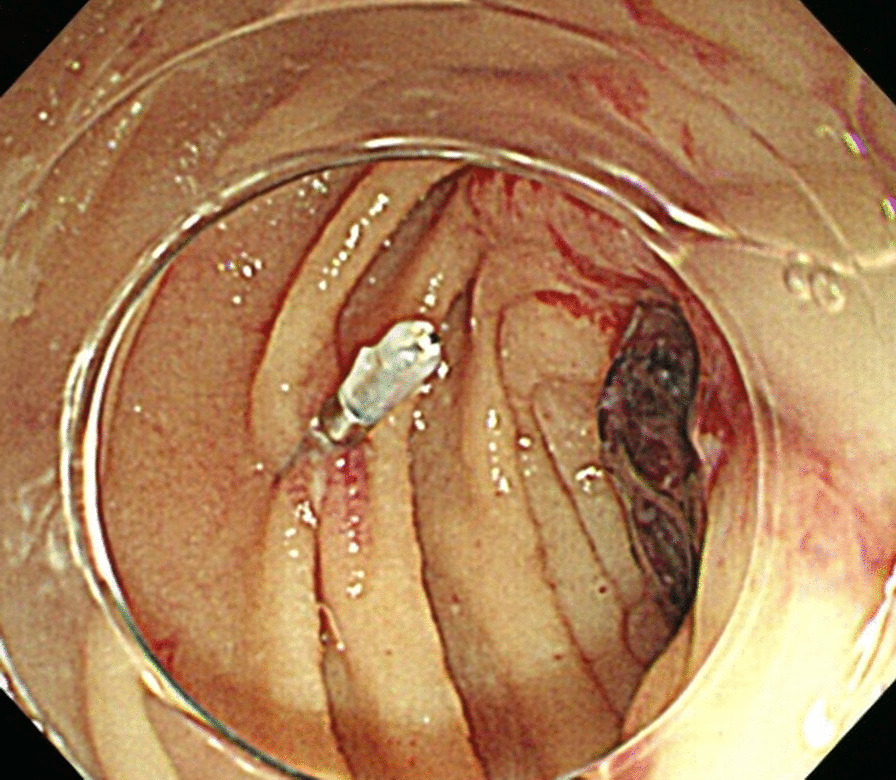
Upper endoscopy (case 5). Pulsatile blood clot in the 3rd portion of the duodenum. A marking clip is placed on the opposite side

**Fig. 8:**
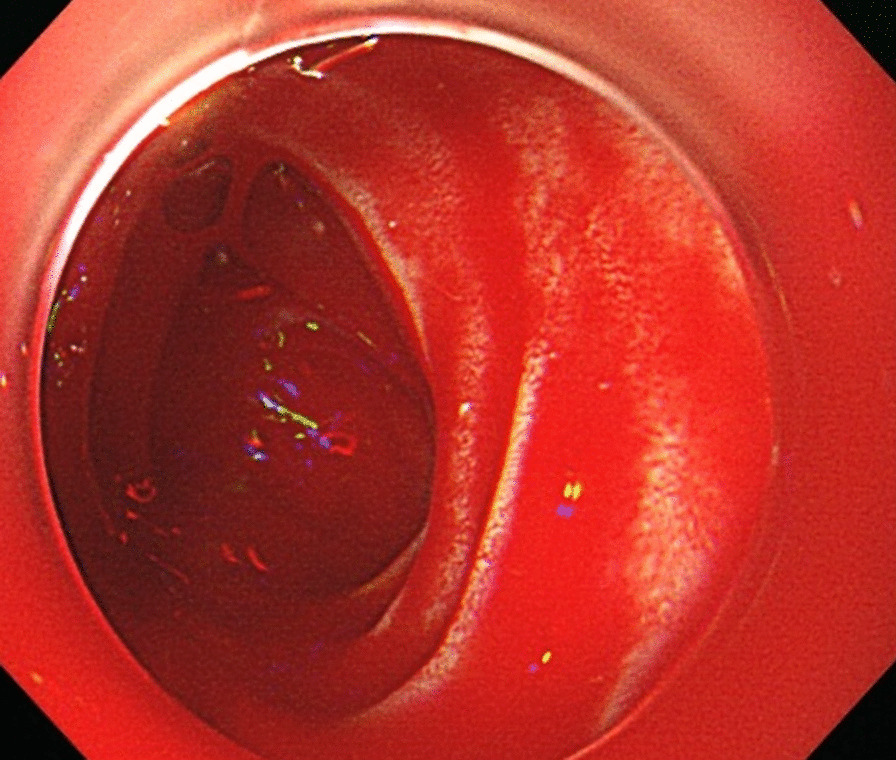
Upper endoscopy (case 4). Massive fresh bleeding of obscure origin is observed up to the 3rd portion of the duodenum

**Fig. 9:**
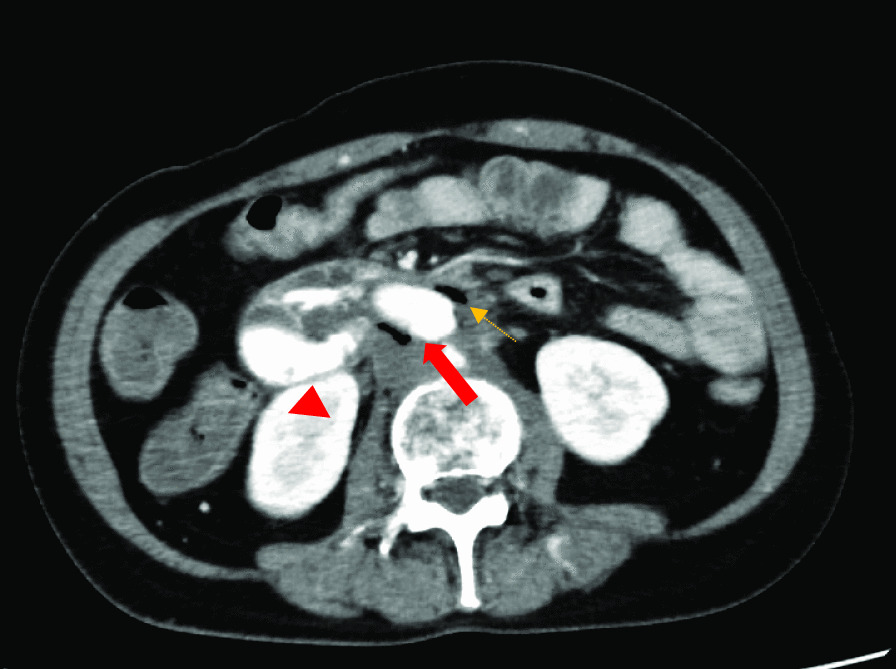
Contrast computed tomography image after an upper endoscopy (case 5). Arrow (solid red)/arrowhead (red): extravascular leakage from the aorta to the duodenum is observed. Arrow (dotted orange): image of air seen in the aorta

**Fig. 10:**
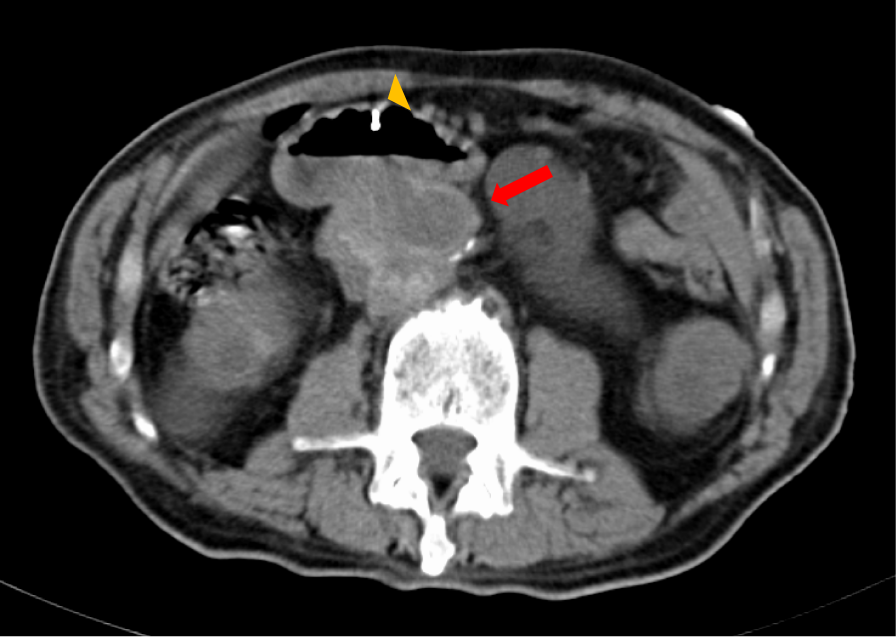
Simple computed tomography (CT) image after an upper endoscopy (case 6). Arrowhead: a cyst-shaped aneurysm adjacent to the duodenum is seen. Arrowhead: since a marking clip was placed on the opposite side, diagnosis of aorto-duodenal fistula was easy, even with a simple CT

The ADF diagnostic procedure is shown in Fig. 1. As the first investigation, upper GI endoscopy was performed on five patients and CT was performed on three patients. When CT was performed first, the aorta and the duodenum were adjacent to each other in all 3 cases (case 1, 7 and 8), and ectopic air was observed in the aorta adjacent to the duodenum in one case (Fig. 2, case 2). However, none of them showed findings of extravascular leakage into the duodenum from the aorta, which is required for the definitive diagnosis of ADF. Therefore, an upper GI endoscopy was performed. Pulsatile protuberances were observed in two patients (cases 1 and 7) (Fig. 3, case 1), and massive fresh bleeding from an unknown origin was found in the duodenum in one case (Fig. 4, case 8). Of the five patients who underwent upper GI endoscopy first, stent exposure was present in only one case, and thus, the definitive diagnosis of ADF was made (Fig. 5, case 2). The other four patients had findings suspicious for ADF. One had a pulsatile protuberance (Fig. 6, case 6), one presented with a pulsatile blood clot (Fig. 7, case 5), and two had massive fresh bleeding from an unknown origin in the duodenum (cases 3 and 4) (Fig. 8, case 4). In these cases, CT was performed after endoscopy. CT showed that the aorta and the duodenum were adjacent in all of these cases; extravascular leakage from the aorta to the duodenum was observed in one case (Fig. 9, case 5), ectopic air in the aorta adjacent to the duodenum occurred in two cases (cases 3 and 5), and a cystic aneurysm in the aorta adjacent to the duodenum was found in two cases (cases 4 and 6) (Fig. 10, case 6). One patient died during endoscopy (case 4), and the diagnosis was confirmed by additional post-mortem imaging. In all these patients, ADF was diagnosed based on both the endoscopic and the CT findings, and the patients were then transferred for surgical treatment. We were able to save the lives of all those patients who underwent surgery.

The fistula site was the horizontal duodenum in seven cases and the descending duodenum in one case. The types of scope used during the diagnosis were GIF-Q260J (Olympus, Tokyo, Japan) in six cases and PCF-PQ260L (Olympus, Tokyo, Japan) in two cases. Although many lesions were observed in the horizontal duodenum, they were visible from the inferior horizontal duodenal angle, which is within the range of the GIF-Q260J. In five cases, the tip attachment was used for upper GI endoscopy, and in some cases, this contributed to the diagnosis. Marking clips were placed in the region of the suspected fistula in four patients, which were very useful in making the definitive diagnosis (Figs. 6 and 10, for case 6).

## Discussion

Amongst the patients diagnosed with ADF during the observation period, only two had shock vitality at the time of visit. However, two of the remaining six patients had shock vitality during endoscopy, and one of them died eventually. It must be recognised that ADF is a disease that causes sudden changes in conditions. However, the patients who could be surgically treated were saved. Prompt and appropriate diagnosis was considered important for improving the survival rate.

Two-thirds of patients with ADF are said to have herald bleeding, transient bleeding before major bleeding [7, 12–15], during which, the presentation of the patient mimics normal upper gastrointestinal bleeding. In fact, we presume that the six patients who presented at the hospital without shock vitality in this study were in this state. In order to save the lives of ADF patients, it is important to make a quick appropriate diagnosis during this period of herald bleeding.

In this regard, the history of aortic intervention is important. All of the cases had undergone intervention for the management of aortic pathology. The average time from aortic treatment to the onset of ADF was 53.4 (7–156) months, and the development of ADF was observed in different periods. Patients with upper GI bleeding and a history of intervention in the aorta should be treated considering the possibility of ADF, regardless of prior treatments. In addition, it is said that infection of the aorta may be a risk factor in the development of ADF. In fact, multiple cases with a high WBC count, CRP level, and body temperature were found and were considered to be infected [16].

In a previous study, findings for making a definitive diagnosis of ADF on endoscopy were reported to be exposure of the artificial blood vessels/stents to the duodenal lumen, and the suspected findings included duodenal clots, extravascular pulsatile tumours, and arterial bleeding from an unknown origin. However, these findings are not necessarily observable, and the rate of diagnosis is 13–38% [3–6]. On the contrary, the pathognomonic finding on CT was the extravasation of blood from the aorta to the duodenum. However, the diagnostic rate of this modality is 30%–61%, which makes it unreliable [4, 8–11]. The findings suspicious for ADF on CT were reported to be loss of continuity of the arterial wall around the duodenum and cystic aneurysm in contact with the aorta/duodenum [7, 8]. However, a previous study reported delayed diagnosis as the above findings were not observed [2]. In the present study, only one case (12.5%) was confirmed as ADF by endoscopy or CT alone, which could be considered inadequate as a diagnostic modality. However, when the two tests were combined, a definitive diagnosis was made, and life-saving treatment could be performed.

The following factors might be helpful when performing endoscopy in these cases: (1) using a tip attachment; in some cases, the fistula site may be hidden behind the folds or flexures, which could be detected by using the tip attachment; (2) converting to a long endoscope as ADF is often present in the horizontal part of the duodenum. This is reflected in our results as well as in a previous report [17], where two cases of ADF were diagnosed using a long endoscope and upon observing the deep duodenum; and (3) using marking clips and performing the CT scan after applying the marking clip, which could make diagnosis of ADF easier (Fig. 6, 10, from case 6).

The limitation of this study is that we were unable to confirm the timing from the onset to endoscopy or CT.

## Conclusions

In a patient presenting with upper gastrointestinal bleeding, a history of intervention involving the aorta should increase the suspicion of ADF. A combination of endoscopy and CT scan is of utmost importance for arriving at a definitive diagnosis. This is especially necessary if there is a chance that infection may complicate the situation. Furthermore, when performing upper GI endoscopy in such patients, tip attachment, converting to a long endoscope, and using a marking clip may aid in the diagnosis.

## Data Availability

The datasets used and/or analysed in the current study are available from the corresponding author upon reasonable request.

## Abbreviations

ADF: Aorto-duodenal fistula
AEF: Aorto-enteric fistula
CT: Computed tomography
